# Lack of Known Target-Site Mutations in Field Populations of *Ostrinia furnacalis* in China from 2019 to 2021

**DOI:** 10.3390/toxics11040332

**Published:** 2023-03-31

**Authors:** Youhui Gong, Ting Li, Xiaojian Xiu, Nicolas Desneux, Maolin Hou

**Affiliations:** 1 State Key Laboratory for Biology of Plant Diseases and Insect Pests, Institute of Plant Protection, Chinese Academy of Agricultural Sciences, Beijing 100193, China; 2 Department of Biological Sciences, Alabama State University, Montgomery, AL 36104, USA; 3 Université Côte d’Azur, INRAE, CNRS, UMR ISA, 06000 Nice, France

**Keywords:** Ace 1 gene, diamide insecticides, genotype, insecticide susceptibility, RyR gene, L1014F mutation

## Abstract

The Asian corn borer, *Ostrinia furnacalis* (Guenée) (Lepidoptera; Pyralidae), is one of the most destructive insect pests of corn, for which chemical insecticides have been the primary method of control, especially during outbreaks. Little information is currently available on the status of insecticide resistance and associated mechanisms in *O. furnacalis* field populations. Invasions and outbreaks of *Spodoptera frugiperda* in China in recent years have increased chemical application in corn fields, which adds to the selection pressure on *O. furnacalis*. This study was conducted to estimate the risk of insecticide resistance by investigating the frequency of insecticide resistant alleles associated with target site insensitivity in field populations of *O. furnacalis*. Using the individual-PCR genotype sequencing analysis, none of the six target-site insecticide resistant mutations were detected in *O. furnacalis* field populations collected from 2019 to 2021 in China. These investigated insecticide resistance alleles are common in resistant Lepidoptra pests and are responsible for resistance to pyrethroids, organophosphorus, carbamates, diamide, and Cry1Ab. Our results support the low insecticide resistance status in field *O. furnacalis* populations and betokens the unlikely development of high resistance mediated by the common target-site resistance alleles. Additionally, the findings would serve as references for further efforts toward the sustainable management of *O. furnacalis*.

## 1. Introduction

Corn (*Zea mays* L.) is a major grain crop in China, and the Asian corn borer *Ostrinia furnacalis* (Guenée) (Lepidoptera: Pyralidae) is one of the primary agricultural pests, frequently causing huge economic costs to corn production [1]. The corn borer larvae damage the ears and stalks by feeding through boring holes, and the damage consequently causes fungal infections or ear contamination, which dramatically decreases the quantity and quality of the corn [2]. Even minor corn borer damage to a corn crop can result in reductions in its market value [3]. The corn yield losses caused by *O. furnacalis* amount to approximately six to nine million tons every year in China [2]. Since resistant corn varieties, including *Bacillus thuringiensis* (Bt) corn hybrids, are not commercially available in China, integrated management measures have been adopted to reduce Asian corn borer infestation levels, including physical control, biological control [4,5], and chemical control. Among them, insecticide application is the primary strategy for *O. furnacalis* control in most of the corn-growing regions, especially during outbreaks [6]. Several classes of insecticides, including pyrethroids, organophosphorus, carbamates, and diamide insecticides, have been employed to control *O. furnacalis* or other pests in the corn crop fields [3,7]. However, extensive use of insecticides may result in harm to the environment and side effects on non-target organisms and on human health, as well as causing insecticide resistance in insect pests [8,9,10,11].

Development of insecticide resistance is common among agricultural pests [12,13,14,15,16]. The high-level insecticide resistance in many agricultural pests is largely mediated by the target site insensitivity due to a single base-pair substitution, while the low to medium level of insecticide resistance is largely due to metabolite resistance mechanisms [12]. Insect species of the same order, or even of different orders, may develop insecticide resistance to the same type of insecticides due to target site insensitivity caused by the same mutations [17]. For instance, mutations of A201S and/or F331W in acetylcholinesterase (AChE, EC3.1.1.7) have been reported to lead to resistance to organophosphorus insecticides, such as triazophos and chlorpyrifos, in many Lepidoptera pest insects, such as *Chilo suppressalis* (pyralidae) [18,19], *Plutella xylostella* (Plutellidae) [20], and *Cydia pomonella* (Tortricidae) [21]. Mutations of G4946E and/or I4790M in the ryanodine receptor (RyR) caused resistance to diamide insecticides in *Spodoptera frugiperda* (Lepidoptera: Noctuidae) [22], *P. xylostella* [23,24], *Tuta absoluta* (Lepidoptera: Gelechiidae) [25], and *C. suppressalis* [26,27]. The knockdown resistance (kdr) mutation L1014F associated with voltage-gated sodium channel (VGSC) insensitivity has been widely reported in several insects, such as *Musca domestica* (Diptera: Muscidae) [28], *Blattella germanica* (Blattodea: Blattellidae) [29], *Culex quinquefasciatus* [30], *Anopheles arabiensis* (Diptera: Culicidae) [31], *Anopheles sinensis* (*Diptera: Culicidae)* [17], *P. xylostella* [32], and *Xenopsylla cheopis* (Siphonaptera: Pulicidae) [33], and has caused resistance to pyrethroids.

Although the insecticide resistance status of *O. furnacalis* is not serious in the field as reported by the Arthropod Pesticide Resistance Database (APRD) [34], the invasion and outbreak of the fall armyworm in China in recent years necessitated extensive application of insecticides for emergency control, which might have exerted selection pressure on *O. furnacalis* [7,35,36]. The problem of insecticide resistance development cannot be ignored in *O. furnacalis.* Unfortunately, almost no other information is currently available on the status of insecticide resistance and associated mechanisms in *O. furnacalis*. Therefore, it is essential to estimate the insecticide susceptibility in field populations of *O. furnacalis*.

In this study, we investigated the presence and frequencies of mutations associated with target-site resistance that have been reported in many pest insects, including the single-pair substitutions A201S and/or F331W in the *AChE 1* (*Ace1*) gene, G4946E and I4790M mutations in the ryanodine receptor, the kdr mutation of L1014F in sodium channel protein para-like, and a 234Y insertion in the ABC transporter subfamily C2 (ABCC2) gene causing Cry1Ab resistance in *Bombyx mori* [37]. The results of this study can help estimate the risk of resistance to insecticides, understand the relative insecticide susceptibility status in the corn borer, and further contribute to sustainable management in the field.

## 2. Materials and Methods

### 2.1. Collection of Field Populations from 2019 to 2021

From 2019 to 2021, a total of 1024 *O. furnacalis* larvae were collected from 12 provinces located principally in southeast and northeast China, crossing the Huanghuaihai summer corn region, the southwest corn region, and the northern spring corn region. One of the populations was collected in 2020 from the experimental plots in Liaoning province, planting the transgenic corn expressing the Bt Cry1Ab protein [38], while all other populations were collected from regular corn fields. The borer samples were individually stored in a 1.5 mL tube with 95% methanol immediately after they were collected from the plants and then stored at −20 °C in the laboratory until use. The field collection details are shown in Table 1.

### 2.2. Individual Crude DNA Extraction

Prior to crude DNA extraction, the collected larvae were taken out from the storage tube and then rinsed individually with distilled water. A small piece of head tissue (no more than 1 cm × 1 cm) was cut from each larva and used in crude DNA extraction.

Crude DNA extraction was performed using a tissue lysate purchased from Mei5 Biotechnology Co., Ltd. (Beijing, China). Tissues were homogenized individually using a stick sharpener in a 1.5 tube containing 20–30 μL tissue lysate. The homogenate was incubated at 95 °C for 5 min in a heater and then centrifuged at 12,000× *g* for 5 min. The supernatant was transferred to a 0.2 mL PCR tube individually and stored at −20 °C until use. The crude genomic DNA extract obtained was used as a template for PCR amplification of a region containing key SNPs that are diagnostic for insecticide mutations as described below.

### 2.3. Examination of Insecticide Resistance Mutations

**Figure 1:**
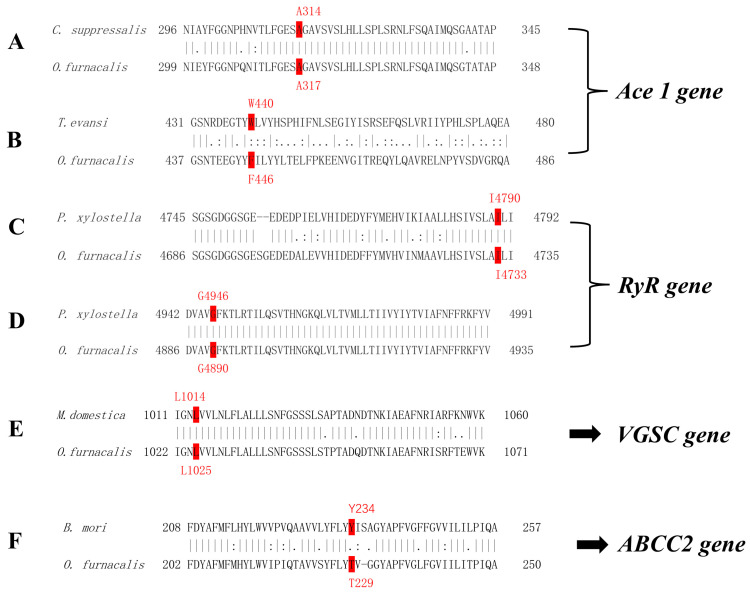
Comparison of the amino acid sequences of target genes between *Ostrinia furnacalis* and other species to locate the amino acid site of resistance mutation. (**A**) For A201S mutation, Ace1 gene amino acid sequence of *O. furnacalis* was compared with *Chilo suppressalis* [18]; (**B**) for F331Y/W mutation in Acetylcholinesterase 1 gene, amino acid sequence of *O. furnacalis* was compared with *Tetranychus evansi* [39]; (**C**) for I4790M mutation in Ryanodine receptors gene (RyR), amino acid sequence of *O. furnacalis* was compared with *Plutella xylostella* [24]; (**D**) for G4946E mutation in RyR gene, amino acid sequence of *O. furnacalis* was compared with *Plutella xylostella* [23]; (**E**) for L1014F mutation in voltage-gated sodium channel (VGSC) gene, amino acid sequence of *O. furnacalis* was compared with *Musca domestica* [28]; (**F**) for 234-site Y insert in ABC transporter (ABCC2) gene, amino acid sequence of *O. furnacalis* was compared with Bombyx mori [37].

In this study, six target-site mutations that have been reported to be associated with insecticide resistance in Lepidoptera insects were investigated. The resistance-related alleles with mutations are listed in Table 2. To precisely design the primers for target alleles, we initially compared the amino acid sequences of target genes between *O. furnacalis* and other species (Figure 1) to locate the amino acid site of mutations. The comparison of amino acid sequences was done at https://www.ebi.ac.uk/Tools/psa/emboss_needle/ (accessed on 15 March 2019). Amino acid site 317 of the *Ace1* gene in *O. furnacalis* corresponds to site 314 in *C. suppressalis* and is equivalent to site 201 in *Torpedo californica*. Amino acid site 446 in the *Ace1* gene in *O. furnacalis* corresponds to site 440 in *Tetranychus evansi* (Acari: Tetranychidae) [39] and is equivalent to site 331 in *T. californica*. Amino acid sites 4733 and 4890 in RyR *O. furnacalis* correspond to sites 4790 and 4946, respectively, in *P. xylostella*, in which G4946E and I4790M mutations associated with diamide resistance were first reported. Amino acid site 1025 in the voltage-gated sodium channel (VGSC) in *O. furnacalis* is equivalent to the 1014-site in *M. domestica*. Amino acid site 229 in ABCC2 in *O. furnacalis* corresponds to the 234-site in *Bombyx mori* (Lepidoptera: Bombicidae), in which the 234-site insertion associated with Cry1Ab resistance was first reported. We also examined this insertion mutation in *O. furnacalis* individuals collected from transgenic corn expressing the Cry1Ab protein in the experimental plots in Liaoning [38] beside field populations collected from 12 sites in China. Based on the amino acid sequences containing the examined mutations, the genomic DNA sequences were addressed to design the primer pairs to amplify the PCR fragments of alleles. The primer pairs for the allele amplification are listed in Table 3. Primer pairs were designed using the Primer-Blast tool on the NCBI database available at https://www.ncbi.nlm.nih.gov/tools/primer-blast/index.cgi (accessed on 20 March 2019).

### 2.4. PCR Amplification and Sequencing

H5 Hiper mix purchased from Mei5 Biotechnology Co., Ltd. (Beijing, China) was used to amplify the DNA fragments of target genes containing the examined resistance mutations using the primers listed in Table 3. The reactions were performed in a final volume of 30 μL with 10 μL H5 Hiper mix buffer, 1.5 μL primer (final concentration was 0.5 μM), 16.5–17.5 μL sterile water, and 1–2 μL of crude DNA homogenate, according to the manufacturer’s instructions. The amplification consisted of 35 cycles (98 °C for 5 s, 60 °C for 20 s, and 72 °C for 15 s), preceded by an initial phase at 98 °C for 30 s and followed by a termination phase at 72 °C for 5 min. PCR fragments were purified and evaluated by DNA gel electrophoresis and then sequenced by Sanger sequencing performed by Sangon Biotech (Beijing, China) Co., Ltd. to get DNA sequences of PCR products. The sequencing primers are also listed in Table 3. The correction of desired sequences was evaluated by using the Basic Local Alignment Search Tool (BLASTn for nucleotide comparisons) available at http://blast.ncbi.nlm.nih.gov/) (accessed on 10 August 2019; 12 October 2019; 1 December 2019), and then the chromatograms of sequences containing mutations were analyzed using ChromasPro software (version 1.62).

## 3. Results

### 3.1. PCR Product Evaluation and Chromatograms of the Insecticide Resistance Mutations

**Figure 2:**
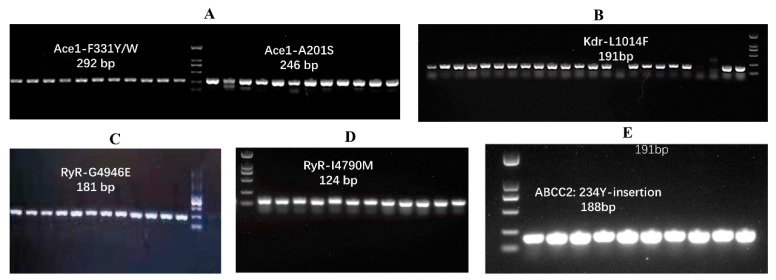
PCR amplification of DNA containing the resistance mutations. We display one figure per mutation with amplification size, which indicates that the DNA fragments containing the corresponding desired mutations were amplified as expected: (**A**) A201S and F331Y/W in Acetylcholinesterase Ace1; (**B**) L1014F in voltage-gated sodium channel (VGSC); (**C**) G4946E in ryanodine receptors (RyR); (**D**) I4790M in RyR; and (**E**) 234Y insertion in ABC transporter (ABCC2).

**Figure 3:**
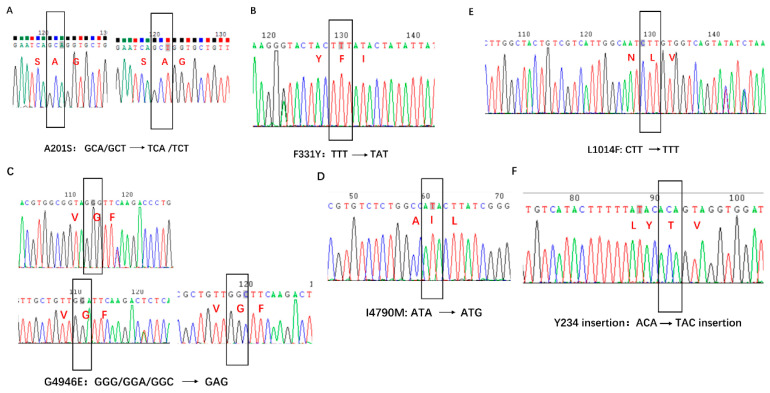
Representative chromatograms of the insecticide resistance mutations examined in Asian corn borer collected in 2019–2021 from China. Black wireframe indicates the site of the examined mutation in the DNA sequence and the nucleotide codon (amino acid) of the examined mutation in all tested samples: (**A**) for the A201S mutation; (**B**) for F331Y/W; (**C**) for the G4946E mutation; (**D**) for the I4790M mutation; (**E**) for the L1014F mutation; and (**F**) for the 234Y insertion.

The DNA fragments of the examined alleles encoding the corresponding desired mutations were amplified in all tested individual samples from 2019 to 2021. Only a single DNA band was amplified in each PCR reaction, and the size of all the amplified DNA fragments was expected to include two DNA fragment lengths of 292 bp and 246 bp responsible for two mutations, F331Y/W and A201S in *Ace1*, respectively; a fragment length of 191 bp responsible for sodium channel mutation L1014F; two fragment lengths of 181 bp and 124 bp responsible for mutations G4946E and I4790M in *RyR*, respectively; and a fragment length of 188 bp responsible for 234Y-insertion in *ABCC2* (Figure 2). The representative chromatograms for each mutation are shown in Figure 3. In all the tested individuals, GCA or GCT encoding alanine was found at 317-site corresponding to the 201-site in *AchE* of *T. californica*, in which the A201S mutation was caused by a single-pair substitution change from GCA/GCT to TCT/TCA (Figure 3A). The expected nucleotide substitution for the Ace1-F331Y/W mutation would be TTT to TAT, but only TTT encoding amino acid phenylalanine was examined in all samples (Figure 3B). A nucleotide substitution from GGG/GGA/GGC to GAG can cause the RyR-G4946E mutation in *P. xylostella*; however, only nucleotide polymorphisms (GGG/GGA/GGC) were found in *O. furnacalis*, which encode amino acid glycine alone (Figure 3C). For the RyR-I4790M mutation, the expected nucleotide substitution is ATA to ATG, but ATA encoding amino acid isoleucine was examined in all tested insects (Figure 3D). For the typical Kdr mutation L1014F based on a nucleotide substitution changing CTT to TTT, only CTT encoding amino acid leucine in the 1025-site in *O. furnacalis* was identified (Figure 3E). The 234Y insertion was examined in all tested samples of *O. furnacalis*, including individuals collected from Bt-corn; however, no insertion of nucleotides encoding tyrosine was found in any tested sample. At the 234-site, the nucleotide codon ACA encoding amino acid threonine was identified in all tested samples (Figure 3F).

### 3.2. Individual Genotype Sequencing Results from 2019 to 2021

A total of 1024 larvae were tested in 2019 and 2021 using PCR genotype sequencing. Based on the chromatogram analysis of each sequence, there was no desired mutation found in any sample. The frequencies of susceptibility of these six resistance mutations were 100% in *O. furnacalis* collected in 2019–2021 from 12 provinces in China, including a population collected from Bt-corn. The genotype of all examined alleles was sensitive homozygote (SS).

## 4. Discussion

Pyrethroids, organophosphorus, carbamates, and diamide insecticides are widely used to control pest insects, including *O. furnacalis* [3,7]. However, the frequent use of insecticides has caused insect pests to develop insecticide resistance worldwide. One of the most important resistance mechanisms is the reduced sensitivity of insecticide target sites, which is caused by the adoption of mutation(s) in the amino acid sequences of target genes [12]. Cases in which insect species of the same order, or even of different orders, developed insecticide resistance to the same type of insecticides due to the target site insensitivity caused by the same mutation(s) have been demonstrated in many studies [17,19,20,21,22,28,30,40]. Although the insecticide resistance status of *O. furnacalis* is not serious in the field, as reported by the Arthropod Pesticide Resistance Database (APRD) [34], little information is currently available on the status of insecticide resistance and associated mechanisms in *O. furnacalis*. Investigating the target-site-insensitivity-associated mutations in this pest could help predict a potential resistance status of field populations. In this study, we, for the first time, tested the insecticide target-site-insensitivity-associated mutations in field populations of *O. furnacalis* from 12 provinces in China using diagnostic PCRs with the sequencing of key genes encoding the desired mutations.

Acetylcholinesterase (AchE) plays a key role in neurotransmission and is the specific target of organophosphate and carbamate insecticides. The mutations adopted in AchE caused a conversion of AchE to an insecticide-insensitive form in several insect species, such as *Drosophila melanogaster* (Diptera: Drosophiladae), *M. domestica*, *Bactrocera oleae* (Diptera:Tephritidae), *Anopheles gambiae* (Diptera: Culicidae), *Culex pipiens* (Diptera: Culicidae), *Myzus persicae* (Homoptera: Aphididae), *Leptinotarsa decemlineata* (Coleoptera: Chrysomelidae), *Cydia pomonella* (Lepidoptera: Tortricidae), *C. suppressalis,* and *P. xylostella* [18,20,40]. Point mutations that confer insensitivity to Ops and carbamates were reported both in *ace1* and *ace2*. This study investigated two typical mutations of A201S and F331Y/W in the *ace1* gene which are found in many pest insects, including *C. suppressalis* [18,19], *P. xylostella* [20], *Apolygus lucorum* (Heteroptera: Miridae) [40], *Bemisia tabaci* (Hemiptera: Aleyrodidae) [41,42], *C. pomonella* [21], *B. oleae* [43], and Tetranychus evansi (Acari Tetranychidae) [39]. However, no mutations in *ace1* were detected from any of the tested samples in this study. This result might be associated with the wide use of the novel diamide insecticides and the gradually decreased use of traditional organophosphate and carbamate insecticides for the control of corn crop pests in China in recent years [5,27]. A reduction in insecticide use may reduce insecticide selection pressure on the pest, consequently reducing the frequency of these mutations in field populations of *O. furnacalis*.

Pyrethroid insecticides have been widely used to control pest insects. Unfortunately, many of the pests have developed resistance to pyrethroids due to mutations associated with VGSC insensitivity. The knockdown resistance (kdr) mutation L1014F (house fly) has been widely reported in several insects, such as *Musca domestica* [28], *Blattella germanica* (Blattaria, Blattellidae) [29], *C. quinquefasciatus* [30], *Anopheles arabiensis* (Diptera: Culicidae) [31], *P. xylostella* [32], and *Xenopsylla cheopis* (Siphonaptera: Pulicidae) [33], among the others. Pyrethroid insecticides are commonly used to control cotton bollworms *Helicoverpa armigera* (Lepidoptera: Noctuidae) and *Agrotis ypsilon* (Lepidoptera: Noctuidae) in cornfields. Interestingly, L1014F was not detected in the field populations of *O. furnacalis* in this study. Additionally, the L1014F mutation is rarely reported in *H. armigera* and *A.ypsilon* [44,45]. This information might indicate low selection pression on this resistant mutation in pests in cornfields.

Diamide insecticides such as flubendiamide and chlorantraniliprole target insect ryanodine receptors (RyR). Diamide insecticides have been widely used in the control of lepidopterans pests, such as *O. furnacalis* and *S. frugiperda,* due to their broad-spectrum high efficacy on numerous pests and excellent safety profile. Diamide insecticide resistance in lepidopteran pests was first reported in *P. xylostella* [23], followed by *T. absoluta* [46] and *C. suppressalis* [26], and very recently found in *S. exigua* [22,47]. The amino acid mutations, G4946E and I4790M, present in the RyR transmembrane domain and causing diamide resistance were first detected in *P. xylostella* [23,48], then in *T. absoluta* [25], *C. suppressalis* [49], and *S. frugiperda* [22]. In our tests of such mutations in RyR in *O. furnacalis*, the frequencies of glutamine in amino acid position 4946 and of isoleucine in position 4790 were 100%, indicating that the insecticide resistance mechanism of G4946E and I4790M mutations in RyR has not evolved in *O. furnacalis*. This result suggests that the development of diamide insecticide resistance caused by G4946E and I4790M mutations have not yet happened in field populations of *O. furnacalis*, and *O. furnacalis* is relatively susceptible to diamide insecticides regardless of metabolic resistance. In another study, Lv et al. [50] reported that the frequency of such mutations in relation to diamide insecticides was very low in 13 populations of *S. frugiperda* in China. In combination, these studies suggest that diamide insecticides are still effective in controlling pests including *S. frugiperda* and *O. furnacalis* in the corn crop fields in China. However, diamide insecticides have been extensively used in recent years to control the populations of the fall armyworm in China [7,36]; the frequencies of G4946E and I4790M mutations need to be further investigated in either *O. furnacalis* or *S. frugiperda.*

Transgenic corn expressing the Bt insecticidal protein has been commercially planted to control the European corn borer *Ostrinia nubilalis* (Hübner) in many countries, and farmers have benefitted from the rapid application of this transgenic technology in the form of reduced insect damage [51]. Although Bt corn hybrids have not been commercially planted in China, many transgenic Bt corn varieties targeting lepidopterans are undergoing regulatory trials, and two varieties of Bt corn were recently issued safety certificates by the Ministry of Agriculture and Rural Affairs of the People’s Republic of China (http://www.moa.gov.cn/ztzl/zjyqwgz/spxx/201912/t20191230_6334015.htm) (accessed on 5 September 2022), which implies that the commercialization of Bt corn may come soon [51]. Li et al. ([51]) investigated the susceptibility and resistance allele frequency of fifteen populations of *O. furnacalis* collected in the Huanghuaihai summer corn region of China to Cry1Ab, Cry1Ac, and Cry1F toxins, and they found that all populations were susceptible to these three Cry toxins and estimated that resistance allele frequency was rare in this region. Liu et al. [52] used the F2 screening method for estimating the expected frequency of resistance alleles in the 13 ACB populations to Bt corn (Bt11 × GA21) expressing the Cry1Ab toxin, and pointed out that the sensitivity of ACB to Cry1Ab was still at a high level, and there were no viable resistant individuals in the field at present. In this study, by directly genotyping the Cry1Ab resistance-associated mutation, we also conclude that a tyrosine (Y) insertion at 234-position in the ABC transporter subfamily C2 (ABCC2) gene was not found in either the non-Bt corn populations (collected from 12 regions in China) or a Bt corn population of *O. furnacalis* that had been subjected to Cry1Ab toxins [38]. This 234-Y insertion in the ABCC2 gene was first reported in *Bombyx mori*, which led to Cry1Ab resistance [37]. Using the CRISPR/Cas9 technique, ABCC2 was also proven to be the functional receptor to Cry1Fa in *O. furnacali* [53]. The findings by Li et al. [51], Liu et al. [52], and this study provide essential knowledge for making the suggestions to commercialize Bt corn, monitor resistance development, and evaluate resistance management strategies in the future in China.

In addition to the use of insecticides to control *O. furnacalis* in cornfields, trichogramma-based biological control has been suggested to be an effective approach for controlling *O. furnacalis* since its use was initiated in the 1970s in China and worldwide [54]. For example, a large-scale release of the parasitoid wasp *Trichogramma dendrolimi* Matsumura (Hymenoptera: Trichogrammatidae) from 2000 to 2015 in the Jilin province, supported by public finances in China, proved to be effective for the control of this pest, as well as led to a decrease in insecticide use across the province [4]. Therefore, with the wide application of Trichogramma-based biological control for the control of the corn borer in China in recent years, it is not surprising that due to the reduction of pesticide selection pressure, no examined insecticide-resistance-associated mutations were identified from the 12 provinces in China sampled in this study.

In total, using the PCR genotype sequencing analysis, we investigated the frequency of six well-known insecticide-resistance-associated mutations corresponding to a range of insecticides, including pyrethroids, organophosphorus, carbamates, diamide insecticides, and Cry1Ab, in field populations of *O. furnacalis* from 12 regions in China. The results show that no targe-site resistance-related alleles were detected. Our results support the low insecticide resistance status of field *O. furnacalis* populations and betoken the unlikely development of high resistance mediated by the common target-site resistance alleles in this pest currently. They also reveal that most insecticides mentioned in this study that correspond to the examined target genes can effectively control *O. furnacalis* in the field. However, other pests in cornfields, such as *S. frugiperda*, should also be taken into account when using insecticides to control *O. furnacalis*. It has been shown that the frequency of G4946E/I4790M mutations in the RyR gene in *S. fru-giperda* is also extremely low [50], suggesting that amide insecticides, whether used to control *O. furnacalis* or *S. frugiperda*, are currently among the most effective ones. However, it should also be used in rotation with other insecticides, taking into account both biological controls to reduce the usage of amide insecticides. In addition, as with similar results observed in other studies [51,52], we did not detect the mutation associated with Bt protein toxins in *O. furnacalis* field populations. This result also lays a foundation for resistance detection of transgenic corn in the near future, once the widespread planting in China has occurred [52]. The metabolic enzymes, such as P450 and carboxylesterase, have been widely studied in insecticide resistance to organophosphate, pyrethroids, and diamide and in the adaptation to contaminants in pest insects [55,56,57,58,59,60,61,62,63,64]. In the future, significant attention should be paid to the metabolic resistance mechanisms of *O. furnacalis* to insecticides, and it remains to be further investigated whether *O. furnacalis* in the field has developed low- to moderate-level resistance to amide insecticides due to metabolic resistance mechanisms.

## Figures and Tables

**Table 1:** Location and number of *Ostrinia furnacalis* samples collected in 2019–2021 in China.

Year	Province and Municipality	Location	Longitude, Latitude	Number Tested
2019	Guizhou	Bijie	105.92° E, 26.84° N	50
	Sichuan	Chongzhou	103.67° E, 30.63° N	50
	Anhui	Bengbu	116.8° E, 33.02° N	60
	Hebei	Hengshui	115.28° E, 37.32° N	45
	Tianjing	Wuqing	117.03° E, 39.22° N	50
	Liaoning	Shenyang	123.56° E, 41.82° N	30
2020	Yunnan	Puer	101.38° E, 22.33° N	25
	Guizhou	Bijie	105.92° E, 26.84° N	49
	Chongqing	Wushan	109.86° E, 31.1° N	30
	Sichuan	Chongzhou	103.67° E, 30.63° N	45
	Jiangxi	Yongxiu	115.81° E, 29.02° N	50
	Anhui	Bengbu	116.8° E, 33.02° N	50
	Jiangsu	Binghai	119.95° E, 34.1° N	45
	Henan	Nanyang	112.8° E, 32.68° N	30
	Shandong	Changqing	116.75° E, 36.55° N	50
	Hebei	Hengshui	115.28° E, 37.32° N	30
	Tianjin	Wuqing	117.03° E, 39.22° N	50
	Liaoning	Shenyang	123.56° E, 41.82° N	35
	Liaoning ^a^	Shenyang	123.56° E, 41.82° N	35
2021	Jiangxi	Yongxiu	115.81° E, 29.02° N	40
	Anhui	Bengbu	116.8° E, 33.02° N	50
	Shandong	Changqing	116.8° E, 33.02° N	40
	Hebei	Hengshui	115.28° E, 37.32° N	50
	Tianjin	Wuqing	117.03° E, 39.22° N	35

^a^: the population was collected in Liaoning province in 2020 from the experimental plots planting the transgenic corn expressing the Bt Cry1Ab protein, kindly provided by Dr. Xueqing Yang (Shenyang Agricultural University).

**Table 2:** The basic information of mutations examined in the present study.

Insecticide Class	Target Gene	Mutations	References
Organophosphorus/Carbamate	Acetylcholinesterase (AchEs)	A201S, F331Y/W	[18,19,20]
diamide insecticides	ryanodine receptors (RyR)	G4946E, I4790M	[22,23,24]
Pyrethroids	voltage-gated sodium channel (VGSC)	Kdr L1014F	[28,30,31]
Bt toxin (Cry Ab)	ABC transporter (ABCC2)	234 site Y insert	[37]

**Table 3:** The primer pairs used for amplification of a region containing key SNPs and the primers used in sequencing.

Mutations	Primer Pairs	Size of PCR Products (bp)	Sequencing Primer
Ace1-A201S	Forward: ATCGTGTTGCATCACTTGGA Reverse: CTGTTGCCGTTCCAGATTGC	246	Forward primer
Ace1-F331Y/W	Forward: CAACAACGAGTGGGGTACCTT Reverse: CTCGAACACTATCGCCTGCC	292	Reverse primer
RyR-G4946E	Forward: GACTGGCGCTACCAAGTGT Reverse: ATGCGTGACAGACTGCAAGA	181	Forward primer
RyR-I4790M	Forward: GAAGTGGTGCACATAGACGAAGA Reverse: GTGATCTCACCTTAAGATGGTAGTACC	124	Forward primer
Kdr-L1014F	Forward: GGAACTTTACAGATTTCATGCACA Reverse: TCTTAACGTTTTTGGTAATCAAG	191	Forward primer
234Y-insertion	Forward: CGGCAAGCTCGTGAATCTTTTG Reverse: CGGCCTGTATTGGCGTTATCAA	188	Forward primer

## Data Availability

All data generated or analyzed during this study are included in this published article.
